# Thermally Conductive Shape-Stabilized Phase Change Materials Enabled by Paraffin Wax and Nanoporous Structural Expanded Graphite

**DOI:** 10.3390/nano15020110

**Published:** 2025-01-12

**Authors:** Yilin Zhao, Shuhui Huang, Zhaoguo Jin, Zhongnan Xie, Hong Guo, Haofeng Xie

**Affiliations:** 1 State Key Laboratory of Nonferrous Metals and Processes, GRIMN Group Co., Ltd., Beijing 100088, China; freewaka@outlook.com (Y.Z.); zhongnanx@126.com (Z.X.); gh_grinm@126.com (H.G.); xiehaofeng@grinm.com (H.X.); 2 GRIMAT Engineering Institute Co., Ltd., Beijing 101407, China; 3 General Research Institute for Nonferrous Metals, Beijing 100088, China; 4 Haiwing Aerospace Materials Research Institute Co., Ltd., Suzhou 215002, China; jinzhaoguo@163.com

**Keywords:** shape-stabilized phase change material, thermal conductivity, latent heat, paraffin wax, expanded graphite, nanoporous structure

## Abstract

Paraffin wax (PW) has significant potential for spacecraft thermal management, but low thermal conductivity and leakage issues make it no longer sufficient for the requirements of evolving spacecraft thermal control systems. Although free-state expanded graphite (EG) as a thermal conductivity enhancer can ameliorate the above problems, it remains challenging to achieve higher thermal conductivity (K) (>8 W/(m·K)) at filler contents below 10 wt.% and to mitigate the leakage problem. Two preparations of thermally conductive shape-stabilized PW/EG composites, using the pressure-induced method and prefabricated skeleton method, were designed in this paper. The expanded graphite formed a nanoscale porous structure by different methods, which enhanced the capillary action between the graphite flake layers, improved the adsorption and encapsulation of EG, and alleviated the leakage problem. The thermal conductivity and the latent heat of the phase-change materials (PCM) prepared by the two methods mentioned above are 9.99 W/(m·K), 10.70 W/(m·K) and 240.06 J/g, 231.67 J/g, respectively, at EG loading by 10 wt.%, and the residual mass fraction was greater than 99% after 50 cycles of high and low temperature. In addition, due to the excellent thermal management capability of PW/EG, the operating temperature of electronic components can be stably maintained at 68–71 °C for about 15 min and the peak temperature can be reduced by at least 23 °C when the heating power of the electronic components is 10 w. These provide novel and cost-effective methods to further improve the management capability of spacecraft thermal control systems.

## 1. Introduction

With the rapidly growing requirements for thermal storage [1,2] for intermittent, transient, and unstable energy sources such as heat dissipation in electronics, the solar industry [3], and industrial waste heat, thermal management technologies based on functional materials have gained much attention. Phase-change materials (PCM) can absorb or release large amounts of latent heat at almost constant temperatures during phase-change processes [4], Therefore, they are widely used in construction, the manufacture of apparel, refrigeration, aerospace, military, communications, and electric power. As a common organic PCM, paraffin wax with high enthalpy (160–270 J/g), non-toxicity, low density, non-subcooling, and stable physicochemical properties is commonly used in low- and medium-temperature thermal storage scenarios. During the operation of spacecraft, electronic components have the characteristics of high-peak heat flow and a narrow operating temperature range, which coincide with the properties of paraffin wax. As a result, paraffin has become one of the most used thermal storage materials (TSM) in spacecraft [5,6,7]. However, the low thermal conductivity of PW, which makes it difficult to meet the increasing heat exchange demand of electronic components, and the difference between the solid and liquid density of PW, which is prone to leakage in encapsulation [8], greatly affects the application of PW in the field of PCMs for thermal storage.

In recent years, significant efforts have been devoted to enhancing the thermal conductivity of paraffin wax by incorporating high-conductivity additives into its matrix. Common thermal conductivity enhancements are mainly divided into the following two categories: metal materials [9,10,11] and carbon materials [12,13,14,15]. Compared with carbon, metal has lower thermal conductivity, and the degree of thermal conductivity enhancement in metal-based PCMs is limited. Therefore, carbon-based composites with excellent thermal conductivity have gained wider attention.

In contrast to low-dimensional carbon nanomaterials, expanded graphite (EG) can be regarded as a derivative obtained from the superposition of multilayer graphite nanoplatelets (GNPs) in aspects of size and structure. EG [16,17,18] is prepared from natural flake graphite through a heating expansion process. The worm-like surface morphology and hollow internal structure of EG, interconnected by GNPs, result in a large specific surface area. This facilitates the phonon vibrations in PW/EG composites, significantly boosting heat transfer efficiency. At the same time, it also increases capillary action [19], which adequately alleviates the leakage issue.

The conventional preparation methods of PW/EG composite phase-change materials are usually divided into two: vacuum impregnation [19,20,21] and melt blending [22,23,24] (Table 1). However, the above methods both introduce free-state EG particles into liquid PW, and due to the difference in density between expanded graphite and paraffin, delamination is inevitable, which leads to inhomogeneous composites. For this reason, the addition of free-state EG in PW improves the overall thermal conductivity of the composites, but the enhancement efficiency is not high. Therefore, achieving higher thermal conductivity (K) (>8 W/(m·K)) with a filler content of less than 10 wt.% and mitigating leakage is still a challenge, so the conventional preparation processes still need to be optimized.

The aim of this study is to prepare PW/EG materials with suitable phase-change temperature, latent heat of phase change, and thermal conductivity that can be used in spacecraft thermal control systems. In this work, we demonstrated two novel methods for the preparation of shape-stabilized paraffin-based thermally conductive enhanced phase-change materials by pressure-induced compression and prefabricated skeleton based on the conventional melt blending and vacuum impregnation preparation methods, respectively. Compared with the PW/free-state EG PCMs in previous studies, in this study, a significant quantity of nanoporous structure was produced by pressing expanded graphite to maximize the thermal conductivity of the composites while minimizing the amount of reinforcement. The above two new preparation processes have their own emphasis on thermal conductivity, heat storage, and leakage resistance.

## 2. Materials and Methods

### 2.1. Materials

Paraffin wax (PW) with a melting point of about 74 °C was used as phase-change material (PCM), and it has a density of 0.85 g/cm^3^ in liquid state, and 1.14 g/cm^3^ in solid state. Expandable graphite powder (50 mesh) is the raw material for expanded graphite.

### 2.2. Preparation of Paraffin Wax/Expanded Graphite Composite

**Figure 1:**
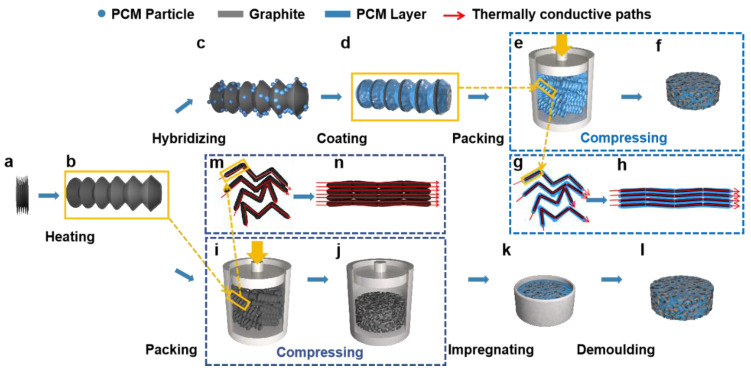
Schematic diagram of pressure-induced method and prefabricated skeleton method.

In contrast to the traditional preparation method of composite PCM, the following two preparation processes of the shaped-PCM: the pressure-induced method (Figure 1c–h) and prefabricated skeleton method (Figure 1i–n) were designed in this work. The specific processes are as follows:

#### 2.2.1. Preparation of Expanded Graphite

During the process of expandable graphite transforming into EG by heating, the intercalated graphite flakes are extended and unfolded along the *x*-axis (Figure 1a,b). EG is primarily prepared through the two methods of high-temperature expansion [25] and microwave expansion [26,27,28]. In this study, the high temperature expansion method was used to obtain expanded graphite by heating the expandable graphite at 900 °C for 30 s.

#### 2.2.2. Preparation of PW/EG Composites by Pressure-Induced Method

Figure 1c–h demonstrate the design concept for the preparation of composite phase-change materials using pressure-induced method obtained by improving the conventional melt blending. The paraffin particles are rotated in a ball mill at 180 rpm for 12 h to obtain paraffin powder. The diameter of crushed paraffin wax is less than 10 μm, which can be well filled into the pores of expanded graphite, and the melted paraffin wax can wet the surface of expanded graphite and form a layer of uniform paraffin film wrapped around the expanded graphite after cooling (Figure 1c,d). Subsequently, the loose PW/EG composite particles are loaded into a mold and pressed into a block (Figure 1e,f). The application of pressure aligns the randomly oriented PW/EG composite particles, creating thermally conductive channels with reduced path lengths (Figure 1g,h). The loading of EG in PW/EG composites ranged from 5 wt.% to 10 wt.%. The sample numbers are shown in Table 2.

#### 2.2.3. Preparation of PW/EG Composites by Prefabricated Skeleton Method

Figure 1i–n present a schematic representation of the prefabricated skeleton preparation method, which serves as an enhancement of the traditional vacuum impregnation technique. To ensure the shaped ability of the composite materials, EG was pressed into a thermally conductive skeleton in advance (Figure 1i,j), and the disordered worm particles were interconnected under directional pressure to form continuous thermally conductive channels (Figure 1m,n). The expanded graphite skeleton and solid paraffin wax were heated under vacuum conditions, where the porous skeleton’s adsorption properties and the pressure gradient across the pores facilitated stronger bonding between the paraffin wax and expanded graphite. The loading of EG in PW/EG composites ranged from 5 wt.% to 10 wt.%. The sample numbers are shown in Table 3.

### 2.3. Characterization

Field emission scanning electron microscopy (JSM-F600, Tokyo, Japan) was employed to examine the morphology of expanded graphite, paraffin powder, and expanded graphite/paraffin-based composite phase-change materials. A powder diffractometer (SmartLab XRD, Tokyo, Japan) was used to characterize the compositions and physical compatibility of pure paraffin, expanded graphite, and expanded graphite/paraffin-based composite PCMs. The scanning speed was 10°/min, and the measured diffraction peaks were mainly concentrated in the range of 15–55°. The functional groups and chemical compatibility of pure paraffin, EG, and PW/EG composite phase-change thermal storage materials were characterized by micro confocal Raman spectroscopy (InVia Qontor, London, UK). The wavelengths ranged from 0 cm^−1^ to 4000 cm^−1^. The specific surface area, pore volume, and pore size of the expanded graphite were measured using a specific surface pore distribution tester (ASAP 2020, Norcross, GA, USA). The latent heat and phase transition temperature of expanded graphite/paraffin-based composite phase-change heat storage materials and pure paraffin wax were analyzed using a differential scanning calorimeter (DSC2500, New Castle, DE, USA) under a nitrogen atmosphere, achieving a measurement accuracy of ±0.01 °C. Each sample weighing about 2–8 mg was placed in an aluminum crucible. The solidification and melting cycles were carried out in the same experimental environment at a cooling and heating rate of 10 °C/min over a temperature range of 25–150 °C. The thermal stability of expanded graphite/paraffin-based composite phase-change materials and paraffin wax was characterized using a simultaneous thermal analyzer (STA 449F, Selb, Germany) under a nitrogen atmosphere. The samples weighed 2–8 mg and were placed in aluminum crucibles at temperatures in the range of 25–300 °C. The thermal conductivity of pure paraffin and composite phase-change heat storage materials was measured using an LFA467 (Selb, Germany) low-temperature thermal conductivity analyzer. The test method used in this instrument is the transient planar source (TPS) method. Three measurements were taken for each sample, and the average value was taken to minimize the error. The IR thermal graphic images were obtained by an infrared camera. The leakage-proof measurements were performed using a high-precision electronic scale, an electric oven, and high and low temperature test chamber.

## 3. Results and Discussion

### 3.1. Characterizations of Morphology and Structure, Physical and Chemical Compatibility

**Figure 2:**
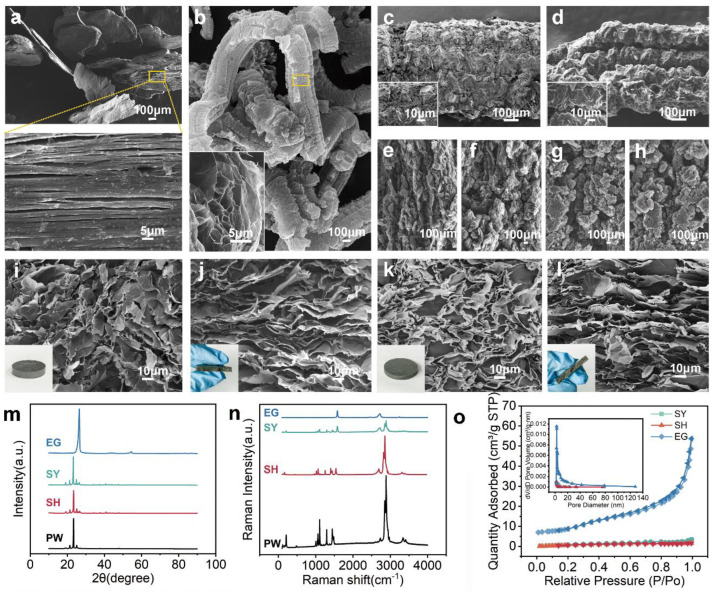
Morphology and structural characterizations of the PW/EG composites. The SEM image of (**a**) expandable graphite, (**b**) EG, (**c**) EG with the PW powder, (**d**) EG with the PW coating, the skeleton of composites with (**e**) 10 wt. % EG, (**f**) 8 wt. % EG, (**g**) 6 wt. % EG, (**h**) 5 wt. % EG, (**i**) transverse section and (**j**) longitudinal section of composites prepared by pressure-induced method, (**k**) transverse section and (**l**) longitudinal section of composites prepared by prefabricated skeleton method. (**m**) XRD and (**n**) Raman spectrum results for EG, PW, and the PW/EG composites, (**o**) Sorption and desorption curves and Pore size distribution of EG skeleton, SH5 and SY5.

The SEM images presented in Figure 2 are as follows: (a) expandable graphite, (b) EG, (c) EG with the PW powder, (d) EG with the PW coating, (e) 10 wt.% EG skeleton, (f) 8 wt.% EG skeleton, (g) 6 wt.%EG skeleton, (h) 5 wt.%EG skeleton, (i) cross-section, and (j) longitudinal section of composite blocks prepared by pressure-induced method, (k) cross-section, and (l) longitudinal section of composite blocks prepared by prefabricated skeleton method. When natural graphite flakes are intercalated, the intrinsic van der Waals bonds are broken, the formation of many tightly packed GNPs are formed (Figure 2a) [29], and natural flake graphite in this state is called expandable graphite. At 900 °C, expandable graphite expanded substantially along the axial direction, with less expansion in the radial direction, generating worm-like expanded graphite after 30 s (Figure 2b). The worm graphite particles have abundant pore structure throughout the body, and the pore structure formed on the surface and inside can be regarded as the graphite nanoplatelets with different orientation connected. In the pressure-induced method, the melted paraffin wax can be easily adhered to the surface of the expanded graphite due to the hydrophobicity of both the graphite nanoplatelets and the PW (Figure 2c,d). In the prefabricated skeleton method, with the addition of different masses of EG, the alignments of the worm-like graphite particles in the expanded graphite skeleton converged to one uniform direction as the EG loading increased at the same pressure (Figure 2e–h). For these two methods, due to the downward pressure exerted, the structures of the composites were all anisotropic (Figure 2i–l). The gap between the graphite flake layer and the PW is slightly ambiguous in the pressure-induced method (Figure 2i), but there is a clear zoning between the prefabricated skeleton and the PW (Figure 2k).

SEM images of expanded graphite before and after pressing (Figure 2) reveal that the unpressed expanded graphite contains large pores that are not at the nanoscale, and the spacing between the neighboring GNPs in the expanded graphite as well as in the cross-section of the composites after pressing has been drastically reduced, forming plenty of nano-porous structure, which can also be confirmed in Figure 2o.

Figure 2m shows the XRD results of EG, PW, and their composites. In the pattern of PW, there are four peaks that represent its crystallization. In the pattern of EG, there are two peaks, the main peak is at 26.44°, representing the (002) strong characteristic peak of graphite. In the pattern of SY and SH, five strong diffraction peaks are shown, which are mainly superimposed on the peaks in PW and EG, but all of them show reduced intensity. Figure 2n shows the Raman results of EG, PW, and their composites. The Raman spectrum of EG features two primary peaks at 1583 cm^−1^ and 2721 cm^−1^, corresponding to the G and G’ bands of graphite, respectively. In addition, SY and SH exhibit all the characteristic peaks of PW and EG, and no new characteristic peaks appear, and none of the peaks are shifted. These results indicate that composite preparation involves no chemical reactions, but rather a physical integration of PW and EG.

Figure 2o shows the pore size distribution results for the EG skeleton, SY (EG loading by 10 wt.%), and SH (EG loading by 10 wt.%). It is observed that the EG, SY, and SH composite blocks all possess mesoporous properties. In comparison to the nanoporous EG skeleton prior to its combination with PW, the composites exhibit a notable reduction in mesoporous pore size (ranging from 2 to 20 nm) and specific surface area, primarily due to the occupation of inter-EG pores by PW. Remarkably, the pore volume of PCM prepared by the pressure-induced method was 2.072 × 10^−3^ cm^3^/g, and PCM prepared by the prefabricated skeleton method was 5.051 × 10^−3^ cm^3^/g. This finding indicates that the graphite structure of the prefabricated skeleton generates capillary resistance during vacuum impregnation, limiting the paraffin wax’s ability to infiltrate the pore space completely and resulting in the formation of voids.

### 3.2. Phase Change Performance and Thermal Decomposition

**Figure 3:**
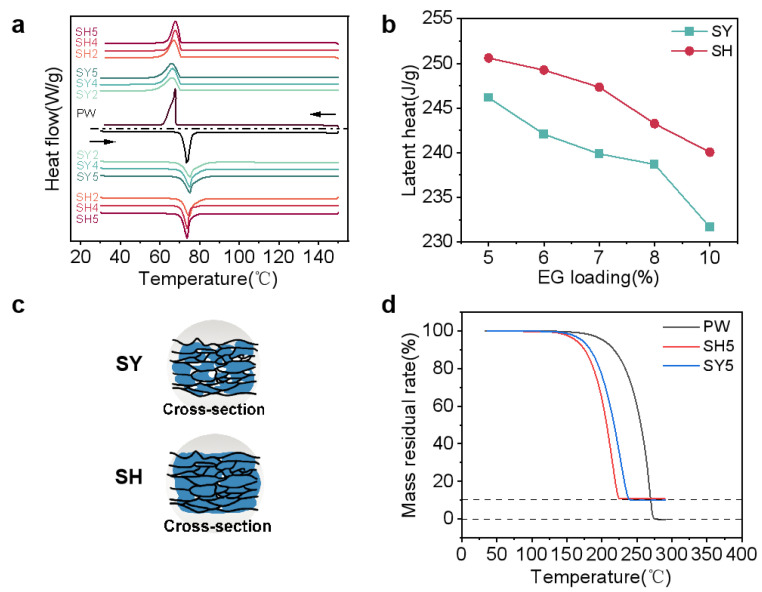
Thermal properties of PW, PW/EG composites. (**a**) DSC curves of different samples; (**b**) comparison of the latent heat of samples prepared by different preparation processes; (**c**) schematic longitudinal sections of samples prepared by different preparation processes; (**d**) thermogravimetric curves of PW, PW/EG composites.

The DSC of the samples PW, SH, and SY are shown in Figure 3 (melting and solidification curves are included), their corresponding phase transition temperatures and the enthalpies are presented in Table 4.

Since the composite material is only a physical mixture of PW and EG, the melting phase-change temperature and solidification phase-change temperature of both PW and PW/EG composites are close to each other in Figure 3a. The latent heat of PW/EG should be linearly related to the mass fraction of PW, which is calculated by the formula ${∆H}_{\mathrm{com}}=\varphi_{\mathrm{PW}}{∆H}_{\mathrm{PW}}$, where $\varphi_{\mathrm{PW}}$ is the mass fraction of PW; the specific values are shown in Table 4. The addition of EG accelerates the heat transfer of the composite phase-change material; therefore, during the heating stage, the phase-change temperatures of EG/PW shift to the right side of the coordinate axis compared to pure PW and shift to the left side during the cooling stage. Among the two preparation processes, the latent heat of PCM blocks prepared by the pressure-induced method is higher (as shown in Figure 3b) for the following reasons: in the prefabricated skeleton process, the paraffin wax could not completely fill the prefabricated skeleton during vacuum impregnation due to the difference in the density of PW between its solid and liquid states and the capillary resistance effect of the graphite, so the composite phase-change material generated more empty pores, which reduced the PW loading, and thus reduced the phase-change enthalpy (as shown in the schematic diagram of the longitudinal cross-section of two composite blocks as shown in Figure 3c). Table 4 presents the BET results for the SY5 and SH5 samples, providing evidence to support the explanation. To meet the heat storage demands of electronic devices, the EG content was regulated within the range of 5–10 wt.%. Notably, the phase transition of latent heat loss for various SY and SH samples did not surpass 13% of that of pure PW.

The thermogravimetric curves of PW and composite blocks are shown in Figure 3d. The weight loss curves of three materials completes in one step of degradation, and the PW starts to degrade by heating at about 150 °C. The mass fraction of the PW/EG composites at complete weight loss is the same as that of the EG reinforcement, and the PW is completely weightless at 275 °C (Table 5).

### 3.3. Thermal Conductivity Enhancement

**Figure 4:**
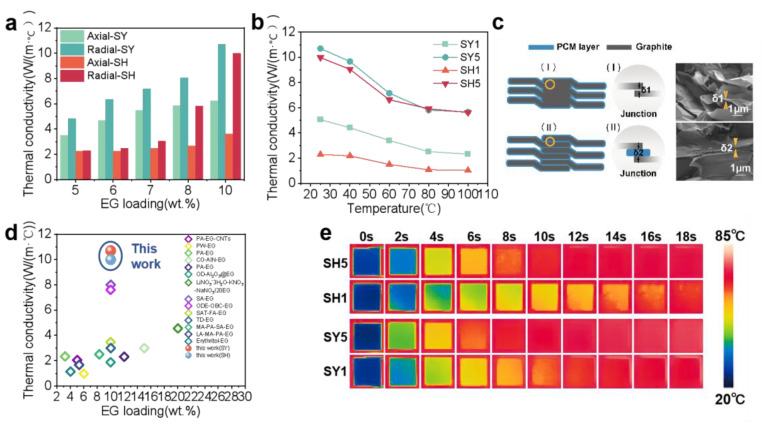
The thermal conductivity of PW/EG composites. (**a**) The radial and axial thermal conductivities of PCMs as functions of EG loading; (**b**) the temperature dependence of radial thermal conductivities of PCMs; (**c**) schematic diagram of explaining the difference in thermal conductivity between the two preparations and SEM images of the longitudinal sections of the two types of material; (**d**) comparison of thermal conductivity of PCMs using different mass fractions of EG as thermal conductivity enhancer [30,31,32,33,34,35,36,37,38,39,40,41,42]; (**e**) infrared images of thermal transport evolution of SH1, SH5, SY1, and SY5 during the heating process.

In contrast to previous methods for preparing thermally conductive phase-change materials, the two techniques further enhance thermal conductivity by altering the orientation of the GNPs and creating a nanoporous structure. However, the force is applied in only one direction, leading to significant anisotropy in PCM. The thermal conductivity for different PW/EG in the transverse (axial) and longitudinal (radial) sections is shown in Figure 4a. The thermal conductivity of the composite blocks improves with the increasing of EG loading, but the radial thermal conductivity of the materials rises significantly faster compared to the axial thermal conductivity and is higher than the axial at the same EG loading. Figure 4c explains the reason that the thermal conductivity of the SY samples is higher than that of the SH samples for the same mass fraction of EG. In the process of the prefabricated skeleton, the expanded graphite particles are pre-pressed into an interconnected 3D mesh structure, and phonon transmission between expanded graphite particles is almost unaffected by the thermal resistance resulting from contact at different interfaces. In the pressure-induced method, paraffin wax obstructs the connection between expanded graphite particles, and the increased interfacial thermal resistance impedes phonon transmission, thereby reducing the thermal conductivity of the composite blocks. The SEM photographs show the longitudinal section of the two types of materials, as we can see, δ1 < δ2, which proves the above viewpoints. At room temperature, the axial thermal conductivities of SY5 and SH5 are 6.23 W/(m·K) and 3.62 W/(m·K), and the radial thermal conductivities are 10.70 W/(m·K) and 9.99 W/(m·K), respectively. Although a large amount of EG can enhance the thermal conductivity of PCM, the thermal storage performance of PCM will be greatly affected, so the EG loading is controlled at 10 wt.% or less.

Considering that there are temperature variations in the electronic equipment of the vehicle during operation, Figure 4b demonstrates the effect of temperature on the thermal conductivity of the PW/EG. It is not difficult to find that the thermal conductivity shows a decreasing trend as the temperature increases, according to the formula for the thermal conductivity, $\lambda =\alpha (T)C_{p}\left(T)\rho (T\right).$ The specific heat capacity changes considerably only after the composites start the phase transition, the density difference of PW in the solid–liquid state is not enough to have much effect on the thermal conductivity, so the thermal diffusion coefficient plays a major role in influencing the thermal conductivity during the temperature change.

As shown in Figure 4e, the composite blocks (8 mm × 8 mm × 2 mm) are placed on a constant temperature heating table at 85 °C. The infrared thermography photographs intuitively show that the better the thermal conductivity of the block, the shorter the heating time is, the faster the heat energy can be transferred through the thermally conductive reinforcing material from the bottom to the top, and the higher the efficiency of the heat transfer.

The expanded graphite formed abundant nanopores by extrusion; the pressure acting in the vertical direction makes the expanded graphite neatly aligned along the radial direction, which shortens the thermal conductivity distance and improves the thermal conductivity efficiency. Compared to paraffin wax, the obtained paraffin wax/expanded graphite composites have about 20 times higher thermal conductivity with less than 13% loss of latent heat.

### 3.4. Shaped Stability and Leakage-Proof Performance

**Figure 5:**
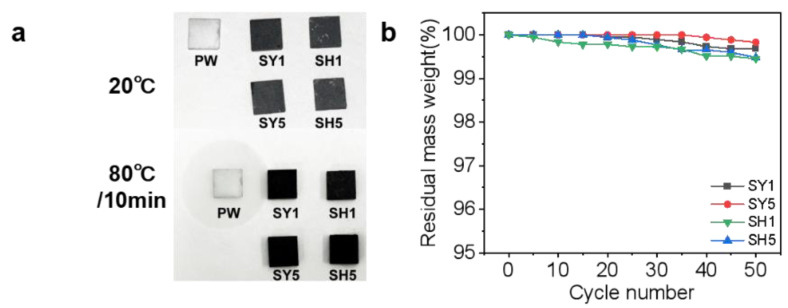
(**a**) Photographs of pure PW, SH1, SH5, SY1, and SY5 at different temperatures; (**b**) residual mass weight of SH1, SH5, SY1, and SY5 over cycle number.

Figure 5a shows the leakage of PW, SY, and SH blocks (8 mm × 8 mm × 2 mm) before and after heating. The unprocessed PW shows obvious melting traces on the filter paper after heating, but the SY and SH composites maintain their original shape without leakage. Figure 5b shows the residual mass of SY, SH composites with different mass fractions of EG undergoing 50 cycles (cycle temperature from 25 °C to 85 °C, one cycle lasting 2 h). The leakage resistance of the PW/EG composites prepared by the two different preparation methods becomes better with the increase in the mass fraction of EG in the composites, but the leakage resistance of the SY samples was stronger than that of the SH samples. In the prefabricated template method, the continuous nanoporous structural EG skeleton has a strong encapsulation ability, and the PW is divided into different small spaces and wrapped by GNPs, which greatly improves the leakage-prone problem of the PW; in the pressure-induced method, the PW can be well adsorbed in the pores of the EG due to the capillary action between the GNPs, but the adsorption capacity of the EG is limited, and the excess PW is still uniformly wrapped around the EG after cooling. Due to the lack of encapsulation or adsorption by the enhancers, the leakage resistance of this part of PW is essentially no different from the unprocessed pristine PW. Both SY and SH samples, as PW/EG composite phase-change materials, were able to maintain stable macroscopic morphology due to the incorporation of EG-shaped reinforcement, and neither of them leaked due to the capillary action in EG. It is noteworthy that the leakage rate of all samples is less than 1% (Figure 5b). In conclusion, both SY and SH samples, as PW/EG PCMs, retain stable macroscopic morphology and demonstrate resistance to leakage due to the inclusion of EG, which functions as a shaped–stabilized reinforcement with a reticulated porous structure.

### 3.5. Thermal Management Test of Electronic Components

**Figure 6:**
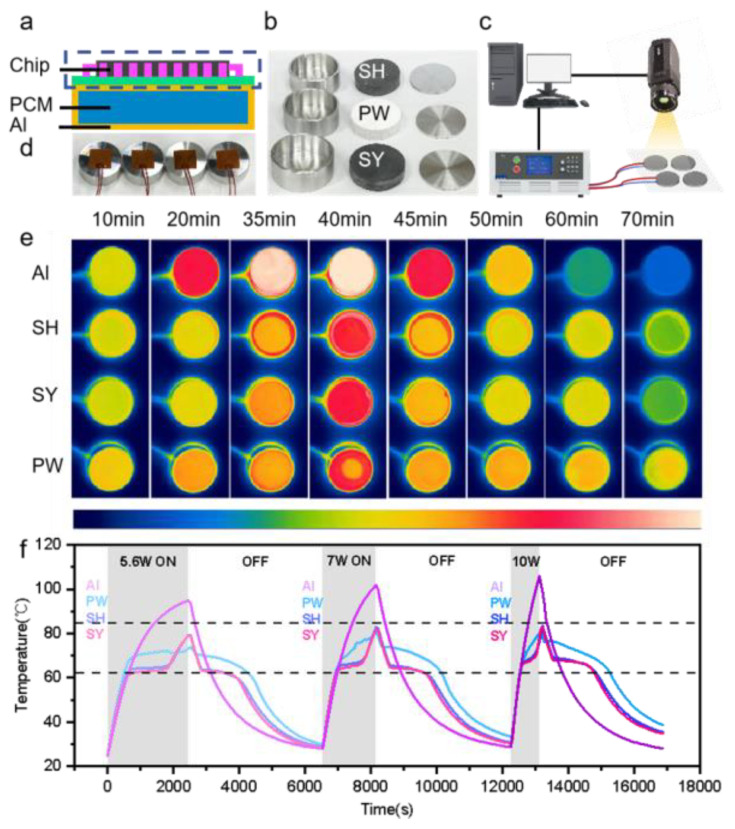
Thermal management test of aluminum shell encapsulated PW, SH5, and SY5. (**a**) Schematic diagram of electronic component and phase-change energy storage panel; (**b**) photographs of phase-change materials encapsulated in aluminum shells; (**c**) schematic diagram of infrared temperature measurement platform; (**d**) photograph of heating pads and aluminum shells encapsulating phase-change materials; (**e**) infrared images of encapsulated blocks during heat storage and exothermic processes from 10 min to 70 min; (**f**) temperature profiles of the encapsulated blocks.

The data processor mounted on an Earth-orbiting satellite is characterized by cyclical work as the satellite rotates, and in response to the heat generation of its equipment, which is characterized by high heat consumption, high heat-flow density, and a narrow design temperature range [43,44,45], phase-change energy storage panels are usually installed around the data processing unit. When the equipment is operating, the phase-change material is used to absorb the heat released by the electronic components to achieve the temperature control requirements, and when the equipment stops running, the phase-change material releases the heat and restores the initial state. A simplified schematic of the electronic components and phase-change energy storage panel is shown in Figure 6a.

In this experiment, a heat storage and exothermic system was built to test the performance of PCMs (Figure 6c). The exothermic effect of electronic components was simulated by using heating pads connected to a constant voltage DC power supply, which were attached to the bottom of the phase-change materials (Φ40 mm, h10 mm) encapsulated by aluminum shells (Φ44 mm, h14 mm, δ2 mm) (e.g., Figure 6b,d), and the power of the heating pads were adjusted by varying the voltage, and then the temperature variations on the surface of the aluminum shells were recorded by a thermal imager. For simplification, the vacuum environment and the intricate structure of the encapsulated housing were omitted, and the size of the PCMs and the power supply were scaled down in proportion to the actual application.

Figure 6e shows the thermal images of the three phase-change materials encapsulated in aluminum shells and the aluminum block as control group at a constant power of 5.6 W. The aluminum block not equipped with PCM has a sensible heat storage method, so it exhibits a very rapid warming and cooling process during both heating and cooling. Compared with the aluminum case loaded with SY5 and SH5, the aluminum case loaded with pure PW only warms up faster, because the thermal conductivity of PW is very low, which seriously affects the heat transfer efficiency between the aluminum shell and the paraffin wax. During the heating process, the sensible heat cannot be converted into the latent heat of the PCM promptly so that the aluminum shell temperature warms up rapidly, and during cooling, the latent heat cannot be converted into the sensible heat of the aluminum case timely, which delays the cooling down process. Compared with the above two materials, PW/EG has a better temperature control ability and can store and release the heat generated by the heating pads in time. As the phase-change materials were not completely sealed by the aluminum shell in this test, it can be observed in the thermal imaging photos of 35 min, 40 min, and 45 min that the leakage of PW is more serious, followed by SH5, and SY5 has almost no leakage.

Figure 6f shows time–temperature plots for four materials at different powers. The first-level derating temperature of the integrated circuit components is 85 °C, and the rated power of the heating pad is 10 W. To maintain the circuit’s safety, the power supply is limited to less than 10 W during the temperature control experiment. Considering the enthalpy of the PCMs and the exothermic power rating of the heating pads, the test utilized phase-change materials with a 20 mm radius and 10 mm height, which were placed inside aluminum shell packages with a 2 mm thickness. The temperature of the aluminum cases was monitored during successive on-off tests of the heater. The aluminum blocks loaded with PW/EG warmed up to about 80 °C after about 40 min, 30 min, and 15 min at 5.6 W, 7 W, and 10 W, respectively, in three cycles.

As the power increases, the heating of the four blocks accelerates and the temperature peaks are higher. At 5.6 W, the peak surface temperatures of the aluminum shells with SH5 and SY5 were 80.33 °C and 80.01 °C, respectively; at 9 W, they were 82.86 °C and 82.05 °C; and at 10 W, they were 83.41 °C and 82.23 °C, respectively. In comparison, the aluminum block without PCM showed a temperature decrease of 15.86 °C and 16.19 °C at 5.6 W; 19.39 °C and 20.20 °C at 9 W; and 23.02 °C and 24.20 °C at 10 W, respectively. According to the curve change, it is not difficult to find that the temperature control ability of PW is poorer compared to the composite blocks filled with EG, although in relation to the temperature of the aluminum block loaded with PW there exists a platform period and the peak value is lower than that of the composite blocks when the heating power is small. Due to the thermal conductivity of PW being quite low, with the increase in the power supply, the heat exchange efficiency between PW and Al is not enough to absorb the heat of the aluminum shell quickly in a short period of time, and it gradually loses the temperature control ability. Comparing the temperature curves of the aluminum blocks loaded with SY5 and SH5, we find that SY5, which has better thermal conductivity, warms up and cools down faster, but the time to maintain the phase-change temperature depends on the enthalpy of the PCM, the temperature plateau period is shorter in SY5 than in SH5, and at the end of three cycles, the surface temperatures of the aluminum shells encapsulated with SH5 and SY5 differed by about 0.3 °C, 0.7 °C, and 0.9 °C, respectively.

## 4. Conclusions

In conclusion, two PW/EG composite phase-change materials with excellent thermal conductivity were prepared by the pressure-induced and prefabricated skeleton methods, respectively. Compared with conventional preparation, changing the orientation of EG by pressure and building a three-dimensional EG thermal conductive network can form a nanoporous structure, which enhances the capillary action, improves adsorption and encapsulation of the expanded graphite, and reduces the thermal resistance. Compared to paraffin wax, the obtained PW/10 wt.% EGs have about 20 times higher thermal conductivity with less than 13% loss of latent heat. The samples prepared by the pressure-induced method have better thermal storage capacity, and the advantages of the samples prepared by the prefabricated skeleton method are mainly shown in the thermal conductivity and leakage-resistant performance. In addition, based on the excellent temperature control performance of PW/EG-shaped phase-change materials, when the operating power of the electronic component using PW/EG with a dimension of Φ40 mm, h10 mm is 10 W, it can be operated stably at 68–71 °C for 15 min, compared to electronic components without a phase-change energy storage panel installed, the maximum peak temperature is reduced by 23 °C. This provides novel methods of PCM synthesis of low cost and a simple preparation process to further improve the management of spacecraft thermal control systems.

## Figures and Tables

**Table 1:** Advantages and disadvantages of the vacuum impregnation and melt blending.

References	Articles	Loading (wt.%)	Thermal Conductivity W/(m·K))	Advantages	Disadvantages
Vacuum impregnation	Yan (2023) [20]	80PW-14EG-6CPEG	4.94	High impregnation rate, less leakage	Complex process, difficult to handle high-temperature PCMs
	Zhong (2009) [21]	92PW-8EG	8		
	Li (2024) [22]	90PW-10EG	2.74		
Melt blending	Zhao (2024) [23]	90PW-5EG-5BC	1.27	Simple process, low cost	Incomplete impregnation
	Yu (2022) [24]	92PW-8EG	2.29		
	Xia (2010) [18]	90PE-10EG	3.83		

**Table 2:** The compositions of different PW/EG composites by pressure-induced method.

Samples	SH1	SH2	SH3	SH4	SH5
Density (g/cm^3^)	0.988	1.009	1.028	1.047	1.103
Mass fraction (wt.%)	5	6	7	8	10

**Table 3:** The compositions of different PW/EG composites by prefabricated skeleton method.

Samples	SY1	SY2	SY3	SY4	SY5
Density (g/cm^3^)	0.946	0.960	0.972	0.987	1.013
Mass fraction (wt.%)	5	6	7	8	10

**Table 4:** DSC results of PW, PW/EG composites.

Samples	Tm (°C)	ΔHm/ΔHcal (J/g)	Ts (°C)	ΔHs/ΔHcal (J/g)
PW	73.40	260.85/260.85	67.68	256.63/256.63
SY1	73.92	246.17/247.81	67.62	242.53/243.80
SY2	74.08	242.07/245.20	66.32	238.04/241.23
SY3	74.68	239.88/242.59	66.26	230.32/238.67
SY4	74.14	238.70/239.98	66.44	229.20/236.10
SY5	74.18	231.67/234.77	66.66	225.48/230.99
SH1	75.85	250.60/247.81	66.26	246.91/243.80
SH2	74.95	249.25/245.20	66.95	245.55/241.23
SH3	74.27	247.34/242.59	67.48	234.88/238.67
SH4	73.92	243.26/239.98	67.71	233.93/236.10
SH5	73.72	240.06/234.77	67.82	231.98/230.99

**Table 5:** The results of specific surface area and pore volume.

Samples	BET Specific Surface Area (m^2^/g)	Total Pore Volume (cm^3^/g)
SY5	2.1882	6.100 × 10^−3^
SH5	2.1090	3.895 × 10^−3^

## Data Availability

The data used are confidential.
